# The Olive and Its Oil

**Published:** 1885-07

**Authors:** 


### The Olive and its Oil.

### CULTIVATION OF THE TREE IN EUROPE—HOW THE OIL IS MADE.

The olive tree is an evergreen, supposed to be a native of Greece, but now naturalized throughout the whole of southern Europe and the northern part of Africa. According to Humboldt the olive is cultivated with success in every part of the Old World where the mean temperature of the year is between 58 and 66 degrees. It attains to the height of about thirty feet, with low branches, very similar in shape to the leaves of the willow, with a soft and bright green color. The upper side of the leaf has precisely that tint familliarly known by the name of olive, but the under side is of a light silvery gray, and as the foliage is wafted by the gentlest breeze, its progress can be traced as it glides across the landscape over the valleys covered with olive gardens, giving it the appearance of a silver cloud. The flowers or blossoms grow in small yellow clusters. The wood, when matured, is of a very hard, brittle and compact nature, color dark red, and susceptible of receiving and retaining an exceedingly brilliant polish, on which account it is extensively used in the manufacture of fancy articles and trinkets. It was also much sought after by the ancients for the carving of their gods and miniature statues. The olive tree was also held by them in great veneration, and the oil was used in pouring out libations at the shrine of their devotions, and at the Olympic games the branches formed the wreaths of the victors. The great duration of the tree is marvellous. Near Terni, in the vale of the cascade of Marmora, is a plantation above two miles in extent, of very old trees, and supposed to be the same plants mentioned by Pliny as growing there in the first century of the Christian era. Some of these in Palestine are estimated at 2,000 years old.

The olive also is a symbol of peace, and excepting the fig leaf was the first leaf spoken of in the Bible—the dove returning to Noah with the olive leaf, "So Noah knew that the waters were abated from off the earth." The olive is generally propagated from shoots, and will thrive best in a dry, rocky or sandy situation. The fruit is a green, oval berry, similar in size and appearance to a green-gage plum, and containing a double-celled nut, and when fresh plucked has a harsh, bitter and disagreeable taste. They are eaten only after having been steeped for several days in a lye of wood ashes, and then pickled in a strong solution of muriate of soda. Olives are principally imported into this country from France and Spain. The oil is the lightest of the fixed oils, and is derived wholly from the fruit. The growth of olives and the manufacturing of the oil affords considerable employment to many of the inhabitants of France and Italy. The time of the olive harvest is always in November and December. The quality of the oil greatly depends upon gathering the fruit in the first stage of maturity—it should be carefully plucked by the hand and the whole harvest completed, if possible, in one day. To concoct the mucilage and allow the water to evaporate, it is spread out for two or three days in beds about three inches deep.

The olive mill is simple. The fruit is reduced to pulp, put into bags or coarse linen or feather grass, and then subjected to pressure. The oil first expressed is finest and sweetest and is termed the virgin oil. A second and afterward a third quality of oil is obtained by moistening the residuum and increasing the pressure. The Tuscans were the first who exported olive oil to any extent, and thus in Europe it is largely known as Florence oil, but the finest and purest virgin oil is said to be obtained from Aix, in France, where are located the extensive olive gardens of the Messrs. La Roux & Sons, whose name has been so long associated with the leading expressers of virgin oil, and whose brand is the conceded favorite with most of the connoisseurs throughout Europe. The varieties of olives are nearly as numerous as the grape or fig—the French alone describe between thirty and forty sorts. The inhabitants of the south of Europe use olive oil for the same purposes as we use butter, and feel at least as much dislike to the product of the dairy as we may feel to the use of oil. In this country it is scarcely eaten, except with salads or for culinary purposes by some classes. Fine virgin oil is also used in all kinds of fine and delicate machines, such as watches, sewing machines, &c. We are told by Malte Brun that if a line be drawn from the Pyrenees through the Cevennes, the Alps and the Haemus, it will separate those countries in which the inhabitants principally make use of butter from those in which they make use of oil. The finest oils are usually imported inro this country in flasks or bottles, which are packed in cases each of one dozen quarts or two dozen pints.

